# Exploring the Multifaceted Biologically Relevant Roles of circRNAs: From Regulation, Translation to Biomarkers

**DOI:** 10.3390/cells12242813

**Published:** 2023-12-10

**Authors:** Parsa Hoque, Brigette Romero, Robert E Akins, Mona Batish

**Affiliations:** 1 Department of Biological Sciences, University of Delaware, Newark, DE 19716, USA; parsa@udel.edu; 2 Department of Medical and Molecular Sciences, University of Delaware, Newark, DE 19716, USA; bmromero@udel.edu; 3 Nemours Children’s Research, Nemours Children’s Health System, Wilmington, DE 19803, USA; robert.akins@nemours.org

**Keywords:** circular RNAs, cardiovascular disease, miRNA sponges, RNA binding protein, gene expression regulation, regulatory axis, biomarkers

## Abstract

CircRNAs are a category of regulatory RNAs that have garnered significant attention in the field of regulatory RNA research due to their structural stability and tissue-specific expression. Their circular configuration, formed via back-splicing, results in a covalently closed structure that exhibits greater resistance to exonucleases compared to linear RNAs. The distinctive regulation of circRNAs is closely associated with several physiological processes, as well as the advancement of pathophysiological processes in several human diseases. Despite a good understanding of the biogenesis of circular RNA, details of their biological roles are still being explored. With the steady rise in the number of investigations being carried out regarding the involvement of circRNAs in various regulatory pathways, understanding the biological and clinical relevance of circRNA-mediated regulation has become challenging. Given the vast landscape of circRNA research in the development of the heart and vasculature, we evaluated cardiovascular system research as a model to critically review the state-of-the-art understanding of the biologically relevant functions of circRNAs. We conclude the review with a discussion of the limitations of current functional studies and provide potential solutions by which these limitations can be addressed to identify and validate the meaningful and impactful functions of circRNAs in different physiological processes and diseases.

## 1. Introduction

In the quest to investigate underlying mechanisms of human diseases, relevant protein-coding genes and their expression have historically been the most well-studied molecular pathways, despite the fact that protein-coding genes comprise less than 2% of the human genome [1]. This means that contributions from much of the human genome composed of noncoding regions remain largely understudied. Increasing evidence over recent years has strongly implicated the potential regulatory roles of these noncoding regions and the resulting noncoding transcriptomic landscape in different biological processes and diseases in humans [1,2,3]. This noncoding transcriptome includes noncoding RNAs (ncRNAs), which are categorized into regulatory and housekeeping ncRNAs. Regulatory ncRNAs include microRNA (miRNA), small interfering RNA (siRNA), piwi-interacting RNA (piRNA), enhancer RNA (eRNA), Y RNA, long noncoding RNA (lncRNA), and circular RNA (circRNA). Housekeeping ncRNAs include ribosomal RNA (rRNA), transfer RNA (tRNA), small nuclear RNA (snRNA), small nucleolar RNA (snoRNA), telomerase RNA (TERC), tRNA-derived fragments (tRF), and tRNA halves (tiRNA) [1,4]. In the context of CVDs and cardiovascular biology, ncRNAs have clinically relevant regulatory roles. For example, both lncRNAs and miRNAs have been recorded to play roles in heart regeneration, remodeling, heart failure, and vascular diseases [3,5,6,7,8,9]. Another class of ncRNAs that includes important regulators of a wide range of biological processes and diseases, including cardiovascular diseases (CVDs), are circRNAs.

The biogenesis, function, and stability of circRNAs have been extensively characterized [10,11,12,13]. In brief, circRNAs are generated from canonical splice sites [12,14,15] via distinct patterns of alternative splicing and back-splicing compared to that of linear isoforms [16]. These splicing events lead to the joining of one downstream splice site to an upstream splice site, a process typically mediated by inverted Alu repeat elements [15,17,18,19,20], thus forming a covalently closed RNA circle. Although previous research shows that circRNA processing and back-splicing form pre-mRNA that is low in efficiency [21], circRNAs can be accumulated at high levels in a cell- and tissue-specific manner [22,23], and have also been observed to be the predominant transcriptional outcome within a wide range of cells [24]. Moreover, circRNAs are notably stable, with a typical half-life of over 48 h, compared to linear mRNAs with a 10 h half-life [25,26], and circRNAs are also resistant to RNAse R and exonuclease-mediated degradation [27,28]. In addition, circRNAs have been observed to act as modulators of cell function by competitively binding endogenous miRNAs and acting as sponges or decoys, inhibiting the activity of miRNAs [29,30]. CircRNAs also act as protein-interactors, affecting many downstream pathways and processes [31,32]. Furthermore, the prevailing assumption of circRNAs being noncoding in nature has undergone a shift, as emerging evidence suggests the potential of some circRNAs to be translated into functional proteins [33,34,35,36]. Their higher stability and multiple biological roles in regulating different pathways have made them attractive candidates for applications as biomarkers and for the study of potential regulatory functions in various biological contexts. Thus far, circRNAs have been revealed to play biologically significant roles in regulating tumorigenesis and metastasis in different types of cancer [37,38,39,40,41], brain development and diseases [42,43,44], bone development and diseases [45,46,47,48], myogenesis and myopathies [49,50], aging [51], and many other diseases and biological processes [52,53,54,55,56,57,58]. Another arena in which circRNAs have recently garnered tremendous levels of functional importance is the regulation of cardiovascular development and diseases [59,60,61,62,63,64]. 

Cardiovascular disease (CVD) is a general term for a group of heart- and blood-vessel-associated conditions including coronary heart disease, strokes, peripheral arterial disease, aortic disease, arrhythmia, valve defects, heart attack, heart failure, and related diseases [65,66,67]. Despite the advancement of strategies to tackle issues related to the diagnosis, prognosis, and treatment of CVDs over the years, the need for clinically relevant and reliable biomarker and novel therapeutic interventions persists to this day [68].

Many different circRNAs have been associated with the regulation of heart function and disease development. The majority of these have been linked to the role of circRNAs acting as sponges or decoys for microRNAs or RNA-binding proteins. Here, we performed a systematic review of the existing literature to identify all the functional roles of circRNAs identified to date. The review begins with an overview of common techniques used for the identification and characterization of circRNAs. We then classified these circRNAs into categories based on the mechanism of action proposed, particularly within the scope of cardiovascular biology and disease [69,70]. 

**Figure 1:**
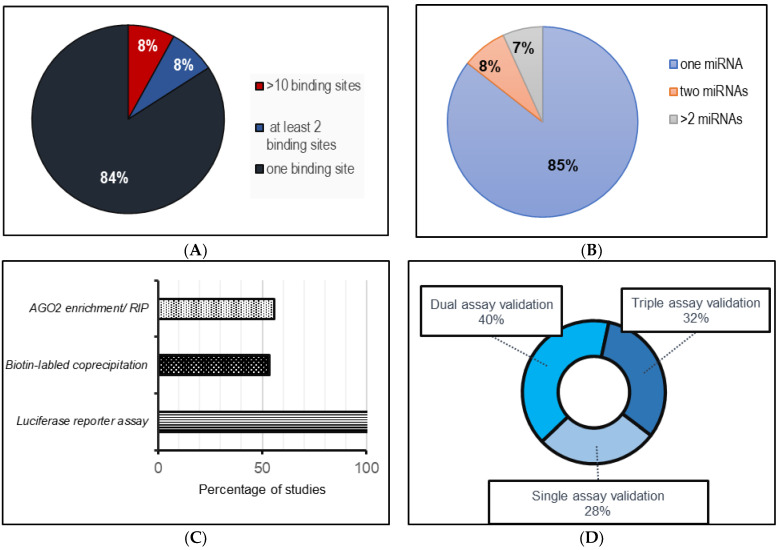
A systematic analysis of the published literature (from 2019–June 2023) involving circRNA–miRNA sponging interactions in cardiovascular systems. (**A**) miRNA binding sites for each miRNA per circRNA; (**B**) number of miRNAs sponged by each circRNA; (**C**) frequency of techniques used to assess circRNA–miRNA interactions; (**D**) method implementation assessment for circRNA–miRNA interactions.

The compilation of data in this study was based on a literature search conducted on PubMed, using filtering parameters spanning from 2019 to 2023. The details of each search strategy and filtering method are included in the text. For example, in the identification of circRNAs that function as miRNA sponges, the following keywords were used: “circRNA-miRNA interactions in cardiovascular system”, “circRNA as miRNA sponges in cardiovascular system”, and “circRNA-miRNA interactions in cardiovascular regeneration”. Studies that did not meet these criteria or did not contain any mention of a clear binding site for an miRNA were excluded. This approach yielded 45 studies of circRNA–miRNA interaction with circRNAs comprising more than one binding site for one miRNA and circRNAs with multiple binding sites for different miRNAs. This analysis is summarized in Figure 1. On the same lines, the selected keywords for circRNA–protein interactions included “circRNA-protein interactions in heart”, “circRNA-protein interaction in cardiovascular system”, “circRNA as protein sponges in heart”, “circRNAs in cardiac regeneration”. The literature for the compilation of circRNAs as biomarkers was carried out using the search term “circRNA biomarkers in heart” using the previously employed search parameters and filters. All research articles and reviews published in English in this time period were included. 

### 1.1. Methodologies for the Identification of circRNAs

**Figure 2:**
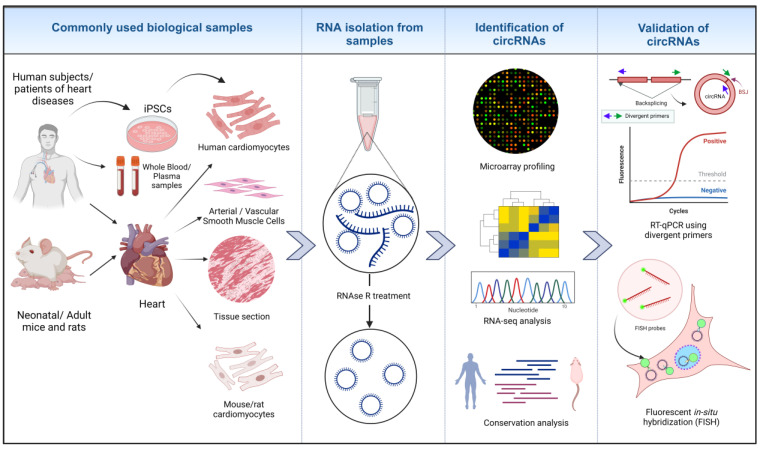
Identification of circRNAs in cardiovascular biology research. RNA is isolated from different biological samples from humans and animal models. RNAse R treatment removes linear RNA species and preserves circRNAs. Micro-arrays, high-throughput RNA-seq technology and analyzing RNA conservation among species leads to the identification of biologically relevant circRNAs. Further validation of identified circRNAs is performed through different assays, such as RT-qPCR and circ FISH [22,26,63] (created with BioRender.com, accessed on 15 November 2023).

In the contemporary studies of circRNAs, total RNA is generally isolated from various biological samples, including from blood/plasma samples, cells, and tissue sections from human and animal models, as depicted in Figure 2. To validate circRNAs, especially novel ones, a common best practice is to use methods like RNAse R treatment to enrich the presence of circular RNAs [71]. Global circRNA detection is typically performed using high-throughput methods such as RNA-seq or microarrays, with either total RNA or r-RNA-depleted RNA and/or RNAse R-treated samples [72]. The RNA-seq is based on the identification and quantification of back-splice junction (BSJ)-spanning reads, and false-positive BSJs can arise from artifacts during library preparation and gene-related issues. For less abundant circRNAs, deep sequencing and longer reads are needed to increase the accuracy of BSJ-mapping [71]. CircRNA profiling from RNAseq is generally carried out using existing annotations available in circRNA databases, including circBASE [73], circRNAdb [74], CircNet [75], and the tissue-specific circRNA database [76]. Additionally, further validation of identified circRNA is often performed through RT-qPCR using divergent primers targeting the BSJ [77] and by imaging using circFISH, allowing for the cellular localization and determination of the copy number of specific circRNAs [78]. 

### 1.2. circRNAs as miRNA Sponges

**Figure 3:**
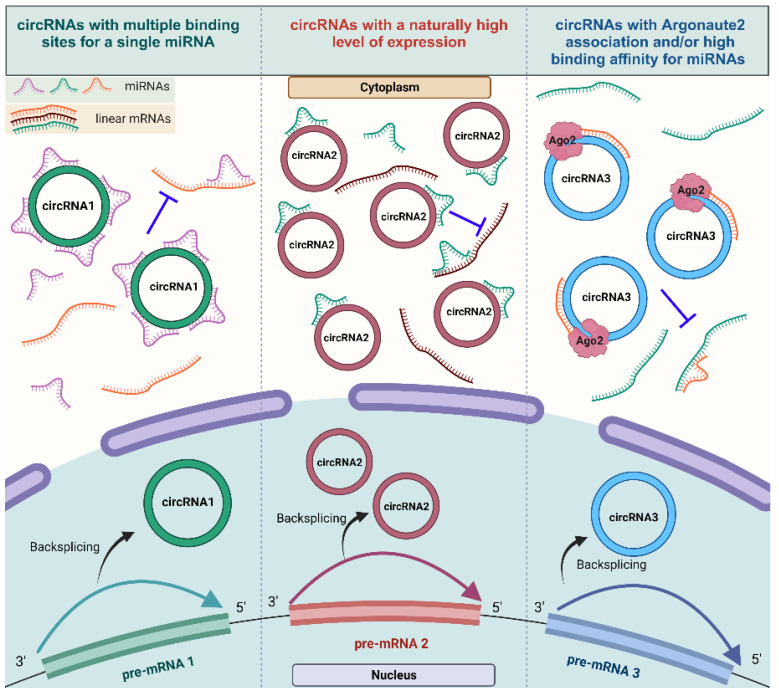
The mechanism of action of circRNAs serving as miRNA sponges. Different types of competent miRNA sponges: circRNAs harboring multiple binding sites for the same miRNA (circRNA1), circRNAs that are expressed at high levels and sponging more miRNAs (circRNA2), and circRNAs with Argonaut 2 enrichment with a strong binding affinity for the miRNA (circRNA3) (created with BioRender.com, accessed on 15 November 2023).

RNA species within both the coding and non-coding transcriptome may contain binding sites for microRNAs (miRNAs), known as miRNA response elements (MREs), allowing for cross-talks between these RNAs as well as a reciprocal influence. Therefore, these RNAs can act as competing endogenous RNAs (ceRNAs), which can regulate miRNA availability [79,80]. A wide range of research investigating circRNAs and their functions has amassed increasing evidence of their ability to “sponge” miRNAs, essentially acting as ceRNAs that suppress miRNA activity and, consequently, increasing the levels of the miRNA targets [12,30,81]. Despite the challenges involved in definitively proving the sponging of miRNA through individual circRNAs [12], the advancement of online bioinformatic tools to assess potential binding sites for circRNA–miRNA interaction, such as Circ Interactome, CircNet, Circ2Traits, and StarBase [29], along with an experimental investigation through RT-qPCR using divergent primers [82], dual-luciferase reporter assays [83], Argonaute (Ago) immunoprecipitation [84], fluorescent in situ Hybridization (FISH) assays [85], and silencing and overexpressing target circRNAs [86], has enabled the discovery of numerous circRNA–miRNA interactions over the past decade. This well-documented function of circRNAs has also garnered recent appreciation in the context of cardiovascular biology and diseases [59,87]. Figure 3 summarizes different scenarios reported regarding the role of circRNAs as miRNA sponges within the contexts of disease, cellular and developmental biology. 

**Figure 4:**
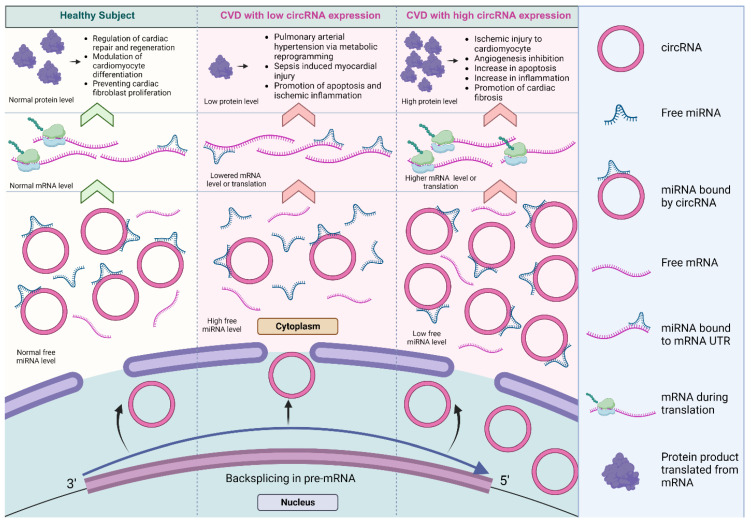
The dysregulation of miRNA sponged by circRNAs in cardiovascular systems. The differential regulation of circRNAs contributed to differences in the level of miRNA inhibition, affecting a variety of biological processes in cardiovascular biology and disease [93,94,95]. The lowered expression of circRNAs results in decreased miRNA inhibition and reduced expression of the target protein. Conversely, the higher expression of circRNAs significantly promotes protein expression (created with BioRender.com, accessed on 15 November 2023).

In the context of the cardiovascular system, several circRNAs have been shown to sponge key miRNAs associated with cardiac development, differentiation, and pathologies [88,89,90,91,92,93]. The differential regulation of circRNAs has been found to contribute to differences in the level of miRNA-mediated inhibition, affecting distinct biological processes in cardiovascular biology and disease [93,94,95], as summarized in Figure 4.

The most well-studied circRNA known to sponge miRNAs is the cerebellar-degeneration-related protein 1 antisense RNA (CDR1as), also referred to as ciRS-7 [30,96]. CDR1as was discovered by Hansen et al. in 2011 as a natural antisense transcript (NAT) derived from the CDR1 gene, resulting from the circularization of the CDR1 antisense exon. The expression of CDR1as was investigated in HEK293 cells and also showed increased enrichment in tissues of the human brain and spinal cord [96,97]. Early studies of CDR1as revealed the presence of 73 selectively conserved miRNA-binding sites for miR-7 in both human and mouse, demonstrating the efficient sponging of miR-7 by CDR1as [30]. As one of the few circRNAs harboring more miRNA-binding sites than expected by random chance, CDR1as holds significant functional importance as an miRNA sponge [22]. The CDR1as/miR-7 pathway was subsequently found to have functional importance in the regulation of post-myocardial infarction (MI) effects in one of the earliest studies of CDR1as’s involvement in cardiovascular disease regulation [95]. CDR1as was found to promote apoptosis in hypoxia-induced cardiomyocytes, and the overexpression of CDR1as resulted in the upregulation of two apoptosis-associated miR-7a targets, PARP and SP1, both in vitro and in vivo. This apoptotic effect was counteracted by the overexpression of miR-7a, resulting in the downregulation of PARP and SP1, and suppression of MI injury induced by CDR1as [95]. 

In contrast to the negative influence of CDR1as in mouse cardiomyocytes, a study conducted on ischemic heart failure in domestic pigs found that the upregulation of CDR1as improved left-ventricle (LV) and right-ventricle (RV) function, with similar trends of miR-7 expression supporting the involvement of the CDR1as/miR-7 pathway in cardiac functional adaptations [98]. In another study involving 30 chronic heart failure (CHF) patients, CDR1as was found to be upregulated in patient plasma and shown to be a potential diagnostic marker [99]. Additionally, CDR1as has also been reported to sponge miR-135a and miR-135b in both primary human cardiomyocytes (hCMs) and the AC16 human cardiomyocyte cell lines. It was found that CDR1as regulated proliferation and apoptosis in these cells by regulating the expression of HMOX1, which is a target for both miR-135a and miR-135b, thus forming two potential regulatory axes in chronic heart failure [99]. Similar experiments in other studies showed that CDR1as promoted calcification in human pulmonary artery smooth muscle cell (HPASMC) in a process regulated by sponging miR-7-5p, which upregulated calcium/calmodulin-dependent kinase II-delta (CAMK2D) and calponin 3 (CNN3) [100]. 

Another key miRNA sponge in the context of cardiovascular disease and development is circular RNA homeodomain-interacting protein kinase 3 (circHIPK3). An early study of circHIPK3 revealed its abundance in the human brain, lung, heart, stomach, and colon tissues [101]. It was discovered to sponge nine different miRNAs, containing a total of eighteen miRNA binding sites in HEK293T cells [101]. Subsequent studies further validated the interaction between the nine miRNAs and cardiovascular diseases in both murine models and human cell lines. For example, miR-29a and miR-29b were both found to be sponged by circHIPK3, regulating cardiac fibrosis [102], diabetic cardiomyopathy [103], and MI [104,105]. Furthermore, circHIPK3′s interaction with miR-124-3p, identified in hCM cells, has been identified as regulating myocardial ischemia/reperfusion (I/R) injury [106]. Additionally, experimental validation has confirmed that circHIPK3 sponges miR-106a-5p, thereby regulating the expression of mitochondrial fusion protein 2 (MFN2) in atherosclerotic (AS) patient tissue, blood samples, and vascular smooth muscle cells (VSMCs). This regulatory network influenced cell mineralization and modulated calcification [107].

Another noteworthy circRNA associated with cardiovascular regeneration after MI is the circular RNA nuclear factor IX (circNFIX). CircNFIX was found to be significantly enriched in the heart of adult mice, rats, and humans, and is regulated by a super-enhancer, where the Meis1 transcription factor binds and positively regulates the expression of circNFIX [93]. In mouse cardiomyocytes, circNFIX was also found to sponge miR-214, harboring three miRNA binding sites, thereby modulating the expression of the cardiomyocyte proliferation and angiogenesis-associated factor, glycogen synthase kinase-3beta (Gsk3β) [93]. CircNFIX sponges miR-145-5p in mouse cardiomyocytes, which upregulates the expression of ATF3, thus inhibiting cardiac hypertrophy [108]. Additionally, circNFIX has been implicated as a potential diagnostic marker, predicting acute ischemic heart disease and sudden cardiac death [109]. 

Other functionally significant circRNAs with multiple miRNA binding sites include circSirt1 [110], circ-calm4 [111,112], circ_Lrp6 [94], and circ-SNRK [113], with the details of their roles summarized in Table 1. The table encompasses research articles that provide insights into multiple binding sites for one miRNA, as well as instances where a circRNA sponges more than one miRNA, irrespective of binding site count, and circRNAs associated with functions other than miRNA sponges.

### 1.3. circRNA-Protein Interactions

**Figure 5:**
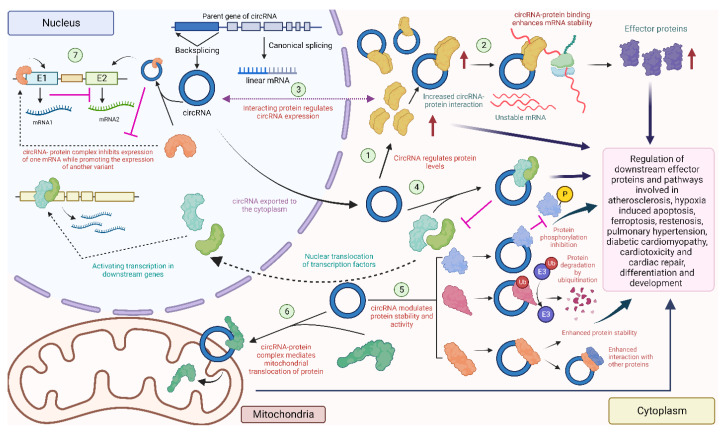
Diverse circRNA–protein interactions in the heart: circRNAs can interact with proteins to regulate their level (1) [135,136], or recruit proteins to enhance mRNA stability and downstream protein levels (2) [137]. Some proteins might affect the expression of the circRNA (3) [115]. CircRNAs can also combine with proteins to prevent nuclear localization of these proteins (4) [138]. CircRNAs can also regulate phosphorylation [139], ubiquitination [140], and enhance the interaction with other proteins [141] by acting as a scaffold (5). Some circRNAs localize in the mitochondria and promote the mitochondrial transport of proteins (6) [142]. Nuclear circRNAs can modulate the expression of downstream mRNAs by interacting with nuclear proteins (7) [127] (created with BioRender.com, accessed on 15 November 2023).

Another significant way circRNAs have been shown to modify cell activities is by interacting with cellular proteins, and a large proportion of the clinically relevant roles of circRNAs are attributed to these interactions. A range of techniques, including RNA binding protein (RBP) immunoprecipitation (RIP), cross-linking immunoprecipitation (CLIP), and electrophoretic mobility shift assay (EMSA), as well as antisense and affinity purification, gradient sedimentation, RNAse protection assay (RPA) and fluorescent in situ hybridization (FISH) combined with immunofluorescence (IF), have revealed a considerable collection of circRNAs that regulate diverse biological pathways by interacting with proteins [31,32]. CircRNAs interacting with proteins have demonstrated the ability to influence endogenous protein interactions, modulate the nuclear and cytoplasmic translocation and/or retention of proteins, and recruit proteins to regulate transcription both positively and negatively. Each of these functional roles have been explored in the context of the cardiovascular system, as depicted in Figure 5. 

Several circRNAs previously known as sponges for multiple miRNAs have also been observed to function as protein interactors. The naturally expressed effective miRNA sponge mentioned earlier, CDR1as, is reported to play a role in diabetic cardiomyopathy (DCM) by modulating the ubiquitination of mammalian sterile 20-like kinase 1 (MST1), leading to the inhibition of MST1 expression and decreased the expression of downstream effector proteins of the Hippo-signaling pathway, including LATS2 and phosphorylated Yes-associated protein (YAP), in DCM mouse cardiac tissues and high-glucose-treated mouse cardiomyocytes [140]. Although the specifics of how CDR1as modulates MST1 ubiquitination remain unexplored in the study, it suggests an indirect interaction with MST1 and possible direct interactions with regulatory factors affecting the ubiquitination of MST1 in the process, thus exerting downstream effects on the Hippo-signaling pathway involved in the pathogenesis of DCM. Furthermore, the downstream effector YAP, associated with DCM, has also been linked to cardiac fibrosis. Another study found that circHelz directly binds to YAP1 and facilitates its nuclear translocation, thereby promoting the growth and proliferation of mouse cardiac fibroblasts (CFs) [143]. Additionally, exons 5 and 6 of YAP pre-mRNA can form hsa_circ_0002320, termed circYap, via back-splicing. This circRNA was found to directly interact with two proteins, Tropomyosin-4 (TMP4) and gamma-actin (ACTG), enhancing their binding to form complexes and regulating cardiac remodeling and fibrosis [141].

Additionally, circHIPK3 was shown to downregulate PTEN in human AC16 cells [135]. It was also found to act as a scaffold to enhance the interactions between the ischemia-associated RBP, human antigen R (HuR) and the E3 ubiquitin ligase, β-TrCP, thereby promoting the degradation of HuR via the ubiquitin–proteasome pathway, leading to cardiac senescence [144]. A foundational study investigating circNFIX in the context of MI showed that circNFIX can promote the degradation of Y-box binding protein 1 (YBX1) through ubiquitination and inhibit its nuclear translocation in rat cardiomyocytes [93]. Additionally, radioimmune precipitation assays and RNA-pulldown assays revealed that altered circNFIX expression influenced the interaction between YBX1 and an E3 ubiquitin ligase, Nedd4l [93]. 

Another notable circRNA in this regard is circFoxo3, as it is reported to be associated with several proteins instead of miRNAs. This predominantly cytoplasmic circRNA was found to interact with cardiac-senescence-associated proteins such as ID1 and E2F1, as well as anti-stress proteins, including FAK and HIF1α, in mouse embryonic fibroblasts. These interactions promote nuclear retention and inhibit their transcriptional activity, as confirmed through pull-down and FISH assays [145]. Additionally, previous investigations in mouse cancer cell lines unveiled that circFoxo3, p21, and CDK2 form ternary complexes, enhancing the interaction between the two proteins while simultaneously preventing the formation of a cyclin E/CDK2 complex. As a result, this disruption impedes cell cycle progression in many cancer and non-cancer cell lines, including mouse CFs [146]. A recent study has also demonstrated that circFox3 inhibits KAT7, which consequently attenuates HMGB1 expression, modulating cardiomyocyte injury, and autophagy in rat H9c2 cells [147]. However, the specific nature of the interaction between circFoxo3 and KAT7 has not been explored to date and requires further validation through RIP, pull-down, and FISH/IF assays.

In addition to being regulated by circRNAs, proteins have also been reported to function as regulators of circRNAs. In addition to the impact of circNFIX on the degradation of the Ybx1 protein, circNFIX expression is driven by the transcription factor Meis1, whose recognition motif resides in the super-enhancer region at the NFIX locus, as studied in mouse cardiomyocytes [93]. Another instance of protein-driven circRNA expression is circBNIP3. The RNA-splicing factor ElF4A3 was found to mechanistically bind to the BNIP3 mRNA and drive the expression of circBNIP3 in hypoxia-induced rat H9c2 cells, serving as an ischemic conditions model for acute MI [115]. ElF4A3 was also discovered to directly bind to circUSP9X in human umbilical vein endothelial cells (HUVECs) in the cytoplasm. This interaction enhances the stability of the pyroptosis-associated GSDMD protein and mediates atherosclerosis (AS) progression [137]. CircKrt4 is another super-enhancer-associated circRNA, which has recently been demonstrated to contain multi-layered interactions with multiple proteins in mouse pulmonary artery endothelial cells (PAECs). This circRNA holds immense potential for therapeutic applications addressing pulmonary hypertension and PAEC injury [148].

Recent evidence highlights the multifaceted protein-interacting capabilities of various circRNAs in the regulation of several biological processes in cardiac cells and tissues. In addition to the circRNAs discussed above and summarized in Table 1, other functionally relevant circRNAs are listed in Table 2. 

### 1.4. Translatable circRNAs in Heart

**Figure 6:**
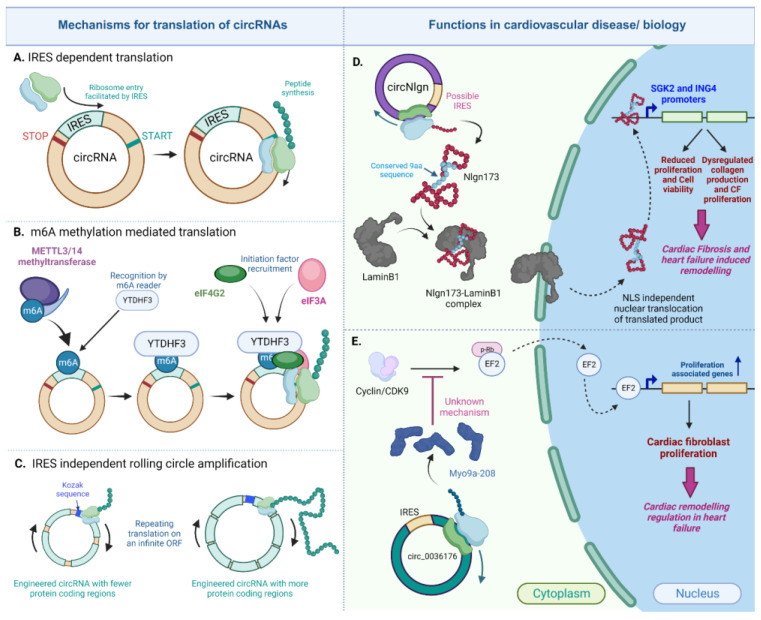
Translated circRNAs in the heart. CircRNAs can be translated in the IRES-dependent, m6A dependent or IRES-independent rolling circle amplification mechanism (**A**–**C**). Two important circRNAs, circNlgn (**D**) and circ_0036176 (**E**), regulate cardiac fibrosis and remodeling in heart failure via their encoded proteins [164,165] (created with BioRender.com, accessed on 15 November 2023).

Despite being traditionally considered noncoding RNAs, there is growing evidence indicating that circRNAs may act as templates for encoding functional peptides [35,161,162]. circRNAs lack the 5′ cap and 3′ polyA tail, which means they need to be translated through a cap-independent mechanism [33,34,163]. Three cap-independent mechanisms have been reported to be utilized by circRNAs to be translated into proteins. Some of these circRNA-encoded proteins have been implicated in functional roles, as outlined in Figure 6. 

One such mechanism is the IRES-dependent translation that is utilized by both eukaryotic and viral transcriptome and its internal ribosome entry site (IRES) for the recruitment of ribosomes to initiate translation independent of the presence of a cap [166,167]. This mechanism is currently the prevailing route of the cap-independent translation adopted by circRNAs to encode peptides [31,34,35,161,162,163]. 

Based on recently published research on translatable circRNAs in the context of the heart, many circRNAs have shown significant associations with ribosomes. For example, van Heesch et al. examined the translational landscape in 80 human heart samples and identified a total of 3181 genes encoding 8878 heart-specific circRNAs [168]. Among these circRNAs, 40 circRNAs encoded from 39 genes were detected to contain associations with ribosome [168], suggesting their potential for translation [168,169]. The list of heart-specific circRNAs included circSLC8A1, circMYBPC3, circRYR2, as well as the very well-established miRNA sponge [96], CDR1as [168]. While the roles of circMYBPC3 and circRYR2 have yet to be investigated, the well-characterized circSLC8A1 was shown to be differentially regulated and exhibited interactions with ribosomes in a human-induced pluripotent stem cell (hiPSC) model for cardiomyopathy (CM), particularly in dilated cardiomyopathy (DCM) [170]. Moreover, circSLC8A1 was proposed as a potential therapeutic target to treat cardiac hypertrophy [171]. A later study attempted to investigate the translational potential of circSLC8A1 by incorporating an in-frame hemagglutinin (HA) tag fused with the circRNA sequence. The tag would only be detectable if the circRNA underwent translation [114]. However, immunoblot results did not confirm the presence of a product of the predicted size from the construct, which suggested that circSLC8A1 might not be translated as previously hypothesized [114]. 

As mentioned in Table 2, circFNDC3B plays an important role in regulating cardiac repair through its interaction with the FUS protein, as was found in mouse cardiac endothelial cells [152]. Although its translation in the context of cardiovascular biology has not been studied to date, another study using human colon cancer (CC) cell lines revealed that the 526-nucleotide-long circFNDC3B contains an IRES sequence within its open reading frame (ORF) [172]. By using four different vectors labeled with a flag, as well as employing Western blotting and LC-MS/MS, the presence of a 218-amino-acid-long peptide was validated and designated circFNDC3B-218aa [172]. Furthermore, the study demonstrated that the encoded peptide had an impact on cellular proliferation, invasion, and migration in CC cells in vitro and in vivo, independent of its parent circRNA. These findings suggest significant clinical implications for the circRNA-encoded peptide [172]. 

A recently identified translatable circRNA in cardiovascular biology is the circular neuroligin RNA (circNlgn), which has been demonstrated to encode the 173 amino acid long peptide, Nlgn173, with functional relevance in cardiac remodeling and cardiac fibrosis [164,173]. In an initial study, Du et al. discovered a novel small protein band of 19kDa in heart and brain lysates from circNlgn-transgenic mice. This protein was found to contain a chain of nine amino acids generated from backsplicing in circNlgn [164]. This amino-acid sequence was found to be conserved across multiple species, and the translation of circNlgn was confirmed through polysome fractionation. Notably, circNlgn was less abundant in the polysome fraction compared to ribosomal subunit fractions obtained from the sucrose density gradient [164]. Further characterization of this novel protein showed its nuclear localization. Despite lacking a canonical nuclear localization signal, the conserved nine amino acid motif naturally interacts with LaminB1 and translocates to the nucleus [164]. Nlgn173 was also found to bind and activate two promoters, SGK2 and ING4, which are essential for the CF fibrosis, proliferation, and survival of cardiomyocytes [164]. In a subsequent study by Xu et al., it was indicated that Nlgn173 binds and activates the DNA repair-associated protein H2AX, leading to its phosphorylation and the activation of downstream effectors involved in cardiac fibrosis [173]. Evidently, circNlgn and its encoded protein emphasize the protein-coding characteristic of circRNAs in the heart. However, the specific mechanism of its translation was not addressed in either study. Although circNlgn was found to be associated with ribosomes, it remains unclear whether this association is due to the presence of an IRES sequence or another mechanism.

Another mechanism closely related to the IRES-dependent process is the translation mediated by N6-methyladenosine (m6A) modification, which is the most abundant base modification found in eukaryotic RNA [174,175]. Previous reports have indicated that human circRNAs containing m6A sites can use these sites as IRESs, facilitating efficient translation. This translation is promoted by the adenosine methyltransferase complex METTL3/14. The translation initiation requires the translation initiation factors eIF4G2 and YTDHF3, which are both part of the family of m6A readers [175,176]. Furthermore, more than 1000 human circRNAs with m6A modifications have been identified in human embryonic stem cells (hESCs) [177], and this enrichment has been observed in other human cells as well [175,176,177]. In fact, in addition to its role in circRNA translation, m6A modification can regulate circRNA biogenesis, degradation, and immunity [176,178,179]. 

The current understanding of the m6A modification of RNAs in the cardiovascular system is limited, but has potential implications in the maintenance of cardiovascular homeostasis [180]. An interesting candidate for the study of translational potential utilizing m6A modification in the context of cardiovascular biology is circZNF609. Previously, circZNF609 has been associated with MI and was shown to be significantly upregulated in the peripheral blood of MI patients, where it was implied to function as an miRNA sponge [181]. Additionally, circZNF609 has been characterized for its functional importance in skeletal muscle myoblast proliferation, showing a heavy association with polysomes and the ability to be translated into a protein through a splicing-dependent mechanism in both humans and mice [182]. As a result of the circularization, circZNF609 formed a 735-nucleotide circular ORF (circORF), which could be translated due to the presence of an IRES located in the circZNF609 UTR, as studied in human and mouse myoblasts [182]. A subsequent study in HeLa cells revealed that the translation of circZNF609 was modulated by m6A modification through the recognition of the initiation factor eIF4G2 and the m6A reader YTDHF3 [175,179]. These studies imply that the translation of circZNF609 is mediated by IRES and enhanced by m6A modification. Additionally, circZNF609 has been reported to interact with YTHDF3 to regulate the expression of YAP in AC16 cardiomyocytes, which was demonstrated to be modulated by m6A modifications, thus promoting heart repair, as listed in Table 2 [149]. Recently, it was discovered that m6A modification negatively affected the stability of circZNF609 and negatively modulated RNA m6A demethylase FTO in doxorubicin-induced cardiotoxicity in neonatal rat cardiomyocytes [183]. Recently, Ouyang et al. reported the functional attributes of the protein encoded by circZNF609, revealing the presence of a functional protein termed ZNF609-250aa. This protein was found to activate the AKT3/mTOR signaling pathway in HK-2 cells and acute kidney injury (AKI) kidneys, affecting cellular autophagy and apoptosis involved in ischemic AKI [184]. However, the role of m6A methylation in the translation of circZNF609 remains unknown and requires further investigation. Given that circZNF609 has regulatory implications and protein interactions occur during cardiac repair, further research is required to characterize the contribution of circZNF609 translation to regulating cardiovascular biology. 

Additionally, there are other mechanisms of circRNA translation that do not require an IRES sequence. Abe et al. were the first to demonstrate that circRNAs synthesized in vitro could be translated without the need for an IRES or similar elements required for internal translation initiation, in both prokaryotic [185] and living human cells [186]. Such translation was mediated by a mechanism like rolling circle amplification (RCA), where this elongation step could continue indefinitely after translation initiation due to the absence of a stop codon [185,187]. Because of the infinite ORF- and RCA-mediated translation, the in vitro-synthesized circRNAs were able to produce proteins in quantities hundreds of times higher than their linear counterparts in HeLa cells [186]. RCA might also induce the production of high-molecular-weight products due to multiple rounds of elongation [185,186,187]. To our knowledge, no translatable circRNAs in the heart have been associated with any such IRES-independent mechanism. Further research is required to characterize translatable circRNAs and their specific mechanisms of translation. 

Taken together, these recent studies suggest that circRNAs, which were previously recognized for their importance in heart function, possess protein-coding abilities. Table 3 summarizes the circRNAs discussed above and other circRNAs involved in cardiovascular biology, along with their potential for translation. The circRNAs in Table 3 were selected based on their implied or experimentally established potential to code functional peptides.

### 1.5. circRNAs as Biomarkers for Cardiovascular Diseases

CircRNAs comprise certain characteristics that enhance their relevance as competent biomarkers. Firstly, circRNAs are stable, with demonstrated resistance to RNAseR and a longer average half-life compared to mRNAs. They also possess disease- and developmental-stage-specific expression profiles [14]. The expression of circRNAs is conserved and abundant across different species [15]. Furthermore, evidence shows that they are not merely accidental byproducts of splicing; instead, they are the predominant transcript isoforms in a wide variety of human cells [24]. CircRNAs can also be present in higher ratios compared to linear mRNAs, as previously reported by whole-blood transcript evaluation, which identified circRNAs as the dominant isoforms [189]. Around 2400 circRNAs were found to be expressed in human blood and exhibited an expression profile like circRNA-rich neuronal tissues [189]. Finally, the cell- and tissue-specific expression of circRNAs make them an excellent choice for biomarkers. They have been shown to play particular roles in the regulation of tissue development and differentiation [76,190,191]. Particularly, the number of circRNAs isoforms varies across different tissues, and in some cases, a circRNA could have a specific function in a particular tissue, while the role of its host gene varies in different tissues [76,192]. Different circRNA isoforms can be expressed due to the alternative back-splicing and the presence of cis- and trans-acting factors involved in circRNA biogenesis [193]. Both alternative 5′ back-splicing and 3′ back-splicing are tissue-specific, with the 5′ type being the most prevalent. The complexity of alternative back-splicing events is positively correlated with intron length and the presence of *Alu* elements and, despite having the same *cis*-elements, different circRNA isoforms could originate from the same host gene (i.e., *MCF2L2* gene) in different tissues, potentially due to *trans*-acting factors [194]. *Trans*-acting factors like RBPs may also modulate the back-splicing pattern of circRNAs in different tissues [136,194,195].

CircRNAs are also abundant in the heart, with thousands of circRNAs expressed in the hearts of humans, mice, and rats [61,63,196]. These characteristics of circRNAs, coupled with their functional significance portrayed in previous sections, position them as promising candidates for cardiovascular disease diagnosis and treatment [197]. 

In addition to the circRNAs with the previously mentioned significant functional roles, other circRNA candidates have been suggested as potential predictors for cardiovascular diseases. In earlier studies, the MI-associated circRNA (MICRA), derived from the ZNF609 gene, was identified as a predictor for left-ventricle (LV) dysfunction following acute MI. The levels of MICRA were assessed in the peripheral blood collected from 642 acute MI patients, and low MICRA levels were associated with high risk for LV dysfunction [198]. In another study involving 136 patients with atrial fibrillation (AF), several circRNAs were detected, among which circ 81906-RYR2 was suggested to be a novel predictor for AF recurrence following surgical ablation [199]. Other circRNAs have also been implicated as biomarkers for postoperative AF (POAF), where hsa_circ_0006314 and hsa_circ_0055387 were evaluated for their potential predictive significance [200]. 

A total of 28 circRNA candidates were identified to circulate in whole-blood samples from 588 cardiac arrest survivors. Among them, the significantly upregulated circNFAT5 exhibited the most potential to predict neurological outcomes and patient survival when used in combination with other predictive biomarkers [201]. Three circRNAs (hsa_circ_0003258, hsa_circ_0051238, and hsa_circ_0051239) associated with lamin A and C proteins in lamin A/C (LMNA) gene-linked dilated cardiomyopathy (DCM), along with one circRNA (hsa_circ_0089762) in ischemic DCM, were significantly overexpressed and suggested to be potential circulating diagnostic biomarkers for etiology-based diagnosis. This conclusion emerged from a study conducted with 20 healthy subjects and 50 DCM patients [202]. 

A recent microarray analysis of exosomes collected from 6 cases of coronary heart disease (CHD) patients and 32 healthy subjects revealed 85 differentially regulated circRNAs, with 4 candidates (circRNA0001785, circRNA0000973, circRNA0001741, and circRNA0003922) being proposed as promising predictive biomarkers for CHD and acute coronary syndrome (ACS) [62]. CircSLC8A1 and circNFIX are another pair of circRNAs relevant to the forensic diagnosis of sudden cardiac death (SCD) and acute ischemic heart disease (IHD). CircNFIX exhibits differential regulation at two different stages of ischemia development [109]. CircRNAs have also shown potential diagnostic utility in heart failure [203,204,205], as well as forensic applications in sudden cardiac death [206]. 

Recent studies conducted since 2019, aiming to investigate certain circRNAs and their differential regulation in cardiovascular diseases and associated physiological conditions, without exploring any other functional attributes, have been compiled and are summarized in Table 4. 

## 2. Discussion

This review reveals the current landscape of circRNA biology in four key domains: miRNA sponging, protein interactions, the expression of circRNA-encoded peptides, and biomarkers. A critical inspection of the studies reviewed in Table 1 raises some interesting questions about specific aspects of circRNAs, such as their use as miRNA sponges. The analysis presented in Figure 2 underscores a significant majority of studies showcasing circRNAs binding to a single miRNA or circRNAs with only one miRNA binding site. Additionally, the expression patterns of most of these miRNAs are negatively correlated with the expression of the apparently sponging circRNAs. This raises a logical concern: if the circRNA indeed sponges miRNAs, total miRNA levels should not be influenced by increasing circRNA expression—a result that has been seen in other studies [127,214]. If miRNAs are indeed negatively regulated, it might be through some intermediate regulatory pathway modulated by the circRNA in the process of miRNA processing and not necessarily through the binding of the circRNA to miRNA, which influences the differential miRNA expression levels [139]. It could also be because of the strong or near-perfect complementarity between the circRNA binding site and the target miRNA, which might trigger the degradation of the miRNA, while an imperfect binding might not result in significant differences in the total miRNA levels [70,215]. These and similar observations resulted in a skeptical view of the sponging activity of the circRNA in question, especially for circRNAs with just one miRNA binding site [69,216]. Given that only a few circRNAs, such as CDR1as and mouse-testis-specific Sry circular transcript, have abundant binding sites for a specific target miRNA [168,217,218], it is challenging to reliably establish that circRNAs with only one binding site have a significant, clinically relevant sponging effect on a particular miRNA target [12,69,70,216]. Some circRNAs, such as circHIPK3, can bind several different miRNAs, although there are only one or two binding sites for each miRNA target [101]. Moreover, circRNAs that can potentially bind more than one miRNA but have few binding sites have been considered weak candidates for effective functioning as miRNA sponges [70]. 

Despite the vast collection of recent studies supporting the sponging effect of circRNAs, as observed in Table 1, it remains unclear how circRNAs with multiple miRNA targets compare to circRNAs with multiple binding sites for a specific miRNA. However, in the context of any circRNA–miRNA interaction study, several criteria must be carefully considered to claim circRNAs as effective miRNA sponges [219]. In addition to evaluating the expression of the circRNA and miRNA, which should be altered enough to produce a sizable biological impact [22], other factors, such as the stoichiometric relationship between circRNA and their target miRNA binding sites, needs to be assessed [12,22,220]. Most of the studies reviewed in this work do not explore the specific details surrounding the interaction between circRNAs and their target miRNAs, which would otherwise be valuable in elucidating precisely how circRNAs influence the regulation of their targets. As seen in Figure 1, luciferase reporter assays were primarily the method of choice to investigate the interaction between the circRNAs and miRNAs in the collected studies. While most of the studies listed in Table 1 validated the circRNA and miRNA binding using at least two validation methods, not all studies explored the potential binding of circRNAs to their target miRNAs through biotin-labeled probe-mediated coprecipitation or by testing for AGO enrichment via RIP or CLIP assays. In this regard, a more thorough examination of the detailed impact of circRNA–miRNA binding is warranted, particularly in terms of its influence on the expression levels of miRNA in response to changing levels of circRNA, the presence of perfect or imperfect complementarity of the miRNA and the circRNA binding site, and any resulting degradation or inhibition [70]. 

Additionally, a more quantitative approach involving the controlled expression systems of miRNA binding sites on circRNAs and the assessment of the relationship between miRNA binding site abundance and activity could shed further light on the stoichiometric relationship between circRNAs and their target miRNAs [220]. It may also prove valuable to investigate the binding affinity of a circRNA to its target miRNA using microscale thermophoresis, as was conducted for the interaction between circ_calm4 and miR-337-3p [112]. It has been suggested that the stronger the binding affinity of a circRNA with its miRNA, the more suitable it is as a candidate as an effective miRNA sponge [70].

In addition to reviewing circRNA sponging activities, we looked for recent literature investigating potential protein interactors in the heart, all of which are compiled in Table 2. We identified several circRNA as interactors with effector proteins within a wide range of cardiovascular contexts. Many of these circRNAs were also previously noted for their miRNA sponging ability, suggesting a multifaceted functional role for these circRNAs. Upon reviewing Table 2, it becomes evident that the majority of circRNA–protein interactions in cardiovascular diseases and developmental biology have been studied in mouse or rat disease models and/or cell lines. A limited number of circRNAs were studied in human cells and tissues. Given the significant functional potential of the circRNAs mentioned above, it would be valuable to explore those circRNA–protein interactions in relevant human cells and tissue models for further elucidation. This exploration could shed light on the relevance of potential human homologs in cardiac development and disease.

Additionally, future studies could benefit from incorporating structural and physical docking visualization and prediction to depict the physical interaction between the circRNA and the protein. Such predictions were validated for the interacting proteins of circKrt4 using the HNADOCK Server [148] and the NPDock server for circYap [141]. Nevertheless, the additional functional attribute of interactions with proteins, combined with the demonstrated potential to sponge miRNAs for many of these circRNAs, clearly amplifies their potential as competent regulatory factors in regulating various aspects of cardiovascular development and diseases. 

Increasing evidence suggests the coding potential of circRNAs, which were previously known to be noncoding [33,34,163,168]. We gathered recent evidence pointing to the potential translatability of circRNAs into functional peptides and their regulatory roles in cardiovascular biology. Notably, circNlgn [164,173] and circ_0036176 [165] were recently validated to play functional roles in cardiac remodeling and fibrosis. Other potential translatable targets for further investigation include circZNF609 [149,182,183] and circFNDC3B [172], as they have been demonstrated to play roles in cardiac repair [183], and MI [152], although their translation in the context of cardiovascular biology remains unexplored. While many of the translation mechanisms rely on IRES-mediated, cap-independent translation initiation, the potential significance of m6A modification, as observed for circZNF609 [149], and its effect on translational efficiency need to be further explored. Currently, it is not understood whether the biological role of circRNAs is affected by the translation mechanism of certain circRNAs. 

We compiled recent evidence showcasing the emerging potential of circRNAs as promising candidates for diagnostic biomarkers for clinical and forensic applications. Although there are several established biomarkers for CVD, many of these biomarkers are influenced by other factors, which limit the possibility of reliable clinical application [221,222,223]. CircRNAs such as circNFIX are abundant, stable, and conserved [93,214], with the additional attribute of having time-dependent differential expression profiles in the development of ischemia [109]. The miRNA sponging activity and protein binding roles of circNFIX, as studied in mouse models [93], not only provide new insights into the function of circNFIX but also encourage further investigation into its relevance in human heart and CVDs. Other circRNAs, such as circCHFR in atherosclerosis [121,124,224], and circ_calm4 in pulmonary hypertension [111,112], consistently exhibit upregulation in their respective diseases and show potential to bind target miRNAs. These circRNAs, along with others sharing similar expression profiles and attributes, stand out as strong candidates for CVD biomarkers. [225,226]. 

CircRNAs are excellent candidates for use as prognostic and diagnostic biomarkers, considering that their half-lives in blood (~24.56 h) are the longest among mRNAs (~16.4 h), lncRNAs (~17.46 h), and miRNAs (~16.42 h). This extended stability increases the time window during which blood samples can be processed [227]. To harness their potential, it is crucial to establish normal baselines that allow for patients to be distinguished from healthy individuals [228]. Additionally, the real clinical application of these biomarkers needs to be further explored by developing independent and multicenter studies that include large population sizes [229,230]. It is also important to note that variation in the expression level of circRNAs could rise due to variables like age, sex, and genetic background, among others [230]. Currently, the implementation of routine screening of specific circRNAs in clinical laboratories poses some challenges due to technical limitations and variability in the pre-analytical phases. The detection of circRNAs could be quite challenging when using plasma or serum due to their relatively low abundance. The relative quantification of circRNAs in whole-blood samples can easily be performed by RT-qPCR, but a standard curve is needed to measure the copy number of circRNAs, making the detection process more complex [231,232]. On the other hand, digital PCR (dPCR) has a higher detection sensitivity, lower sensitivity to inhibitors, and provides absolute quantification of circRNAs, omitting reference genes for quantification [231,233]. Even low-expressed circRNA could easily be detected in saliva, urine, and plasma by using dPCR [231,234]. Droplet digital PCR (ddPCR) is emerging to show great promise in clinical diagnosis due to its higher sensitivity, reproducibility and accuracy [234,235,236] but is still not widely used due to its high cost and complex data analysis. Overall, there is a need for standard operating procedures and guidelines that ensure the reproducibility of the results [229,230]. 

In conclusion, we believe that this comprehensive review serves to communicate the current understanding of the functional roles of circRNAs in cardiovascular biology and highlights specific circRNAs that warrant further investigation. Additionally, we addressed key considerations regarding potential challenges in conventional circRNA research, which can guide future studies to enhance the robust and effective characterization of circRNAs associated with the heart and CVD. 

## Figures and Tables

**Table 1:** CircRNAs as miRNAs sponges in cardiovascular disease.

circRNA	Original Gene	miRNA(s) Sponged/mRNA Target	Number of miRNA Binding Sites	Disease/Biological Context	Biological Sample	circRNA Expression	miRNA Expression	Refs.
CDR1as	Cerebellar-degeneration-related protein 1 (CDR1) exons	miR-7/PARP and SP1	73	MI	Mouse tissue and cardiomyocytes	Upregulated	Upregulated	[95]
		miR-7-5p/CAMK2D and CNN3	8	Pulmonary hypertension (PH)	HPASMCs	Upregulated	Decreased	[100]
		miR-135a and b/HMOX1	1	CHF-associated proliferation and apoptosis	CHF patient plasma, HCMs, and AC16 cell lines	Upregulated	Decreased	[99]
CircHIPK3	Homeodomain-interacting protein kinases (HIPK3) exon 2	miR-106a-5p/MFN2	1	Atherosclerosis	AS patient tissue, blood samples, and VSMC	Low expression	High expression	[107]
		miR-29a/IGF-1 and VEGF	1	MI	Mouse cardiac microvascular endothelial cells (CMVECs)	Upregulated	Upregulated	[104,105]
		miRNA-124-3p	1	Myocardial ischemia/reperfusion (IR) injury	HCM cells	Highly expressed	Negatively correlated with circRNA expression	[106]
circNFIX	Nuclear Factor IX exon 2	miR-214/Gsk3β	3	MI and cardiac regeneration	Mouse cardiomyocytes	Overexpressed in human, rats and mice	Negatively correlated with circRNA expression	[93]
		miR-145-5p/ATF3	1	Cardiac hypertrophy	Patient plasma, neonatal mouse cardiomyocytes, mouse model	Downregulated	Negatively correlated with circRNA expression	[108]
circSlc8a1	Sodium–calcium exchanger gene Slc8a1	miR-133a	17	Cardiac hypertrophy	Neonatal mouse cardiomyocytes	Unchanged during myocardial stress response, but overexpression leads to heart failure	Unaffected by circRNA expression and lower expression is associated with disease development	[114]
circ_Lrp6	Lipoprotein receptor 6 (Lrp6)	miR-145/ITGβ8, FASCIN, and KLF4	At least 7	VSMC growth, differentiation, and homeostasis	Mouse and human VSCMs	Enriched in VSMCs	Not correlated with circRNA expression	[94]
circ-SNRK	Sucrose nonfermenting 1-related kinase (SNRK) exon 1-2	miR-33/SNRK	7	Heart failure (HF) and associated hypoxia	Primary neonatal rat cardiomyocytes and HF rat model	Decreased	Unchanged	[113]
Circ_BNIP3 (hsa_circ_0005972)	BCL2 interaction protein 3 (BNIP3) gene chr10:133784141-133787447	miR-27a-3p/BNIP3, and 2 more unexplored miRNAs, miR-27b-3p and miR-128-3p	1	Hypoxia induced cardiac myocyte injury in ischemia and acute myocardial infarction (AMI)	Rat H9c2 cells	Upregulated	Downregulated while the other 2 were unchanged	[115]
circTRRAP (hsa_circ_0081241)	Transformation/transcription domain-associated protein (TRRAP) gene chr7:98495363-98506585	miR-761/MAP3K2	1	Hypoxia induced cardiomyocyte injury in AMI	AC16 human cardiomyocytes	Overexpressed	Downregulated	[116]
		miR-370-3p/PAWR	1	Hypoxia induced cardiomyocyte injury in AMI	AC16 human cardiomyocytes	Upregulated	Negatively correlated	[117]
CircRbms1 (mmu_circ_0001022, hsa_circ_0002136)	RNA binding motif single-stranded interacting protein 1 (Rbms1) gene	miR-742-3p/FOXO1	1	Hypoxia induced cardiomyocyte injury in MI	MI mouse model tissues and mouse cardiomyocytes (H9c2)	Upregulated	Negatively correlated with circRNA expression	[118]
		miR-92a/BCL2L11	1	AMI	MI mouse model and mouse cardiomyocytes (H9c2)	Overexpressed	Decreased	[119]
		miR-2355-3p/MST1	1	I/R injury	H/R induced HCMs	Increased	Decreased	[120]
circCHFR (circ_0029589)	Checkpoint with forkhead and ring finger domains (CHFR) gene chr12:133428203-133430159	miR-15b-5p/GADD45G	1	Atherosclerosis	ox-LDL) induced HUVECs	Upregulated	Downregulated	[121]
		miR-424-5p/IGF2	1	Atherosclerosis	Ox-LDL-treated human VSMCs	Upregulated	Downregulated	[122]
		miR-214-3p/Wnt3	1	Atherosclerosis	Ox-LDL-treated human VSMCs	Upregulated	Negatively correlated	[123]
		miR-370/FOXO1	1	Atherosclerosis	Ox-LDL-treated human VSMCs	Upregulated	Negatively correlated	[124]
circArhgap12	Rho GTPase activating protein 12 (ARHGAP12) gene exon 3 and exon 2	miR-135a-5p/ADCY1, and 7 more miRNAs	1	Doxorubicin-induced cardiotoxicity	Mouse cardiomyocytes	Upregulated	Upregulated while 7 others were unchanged	[125]
		miR-630/EZH2	1	Atherosclerosis	Mouse aortic smooth muscle cells (MASMCs)	Upregulated	miR-630 was negatively correlated and miR-610 was unchanged, but not used for sponging validation	[126]
circSMOC1	Modular calcium-binding protein 1 (SMOC1) gene exon 4 to exon 7	miR-329-3p/PDHB	1	Pulmonary hypertension	Rat pulmonary artery smooth muscle cells	Downregulated	Unchanged	[127]
circSirtuin1 or circSirt1	Sirtuin 1 (SIRT1) exon 2 to exon-7	miR-132/212/Sirt1	3	Atherosclerosis	Human and rat arterial tissues and VSMCs	Low expression	Negatively correlated with circRNA expression	[128]
		miR-145-5p/Akt3	1	Pulmonary hypertesion	Rat model for PH and Human PASMCs	Increased	Decreased	[110]
circ-calm4	Calmodulin 4 gene single exon	miR-337-3p/Myosin 10	17	Pulmonary hypertension	Mouse pulmonary artery smooth muscle cells (PASMCs)	Upregulated	Downregulated	[112]
		miR-124-3p/PDCD6	2	Pulmonary hypertension and vascular smooth muscle cell pyroptosis	PH mouse model and cultured pulmonary artery smooth muscle cells (PASMCs)	Upregulated	Downregulated	[111]
circ_0010283	Ubiquitin protein ligase E3 component n-recognin 4 (UBR4) gene chr1:19449326-19480433	miR-370-3p/HMGB1	1	Atherosclerosis	ox-LDL-induced HVSMCs	Upregulated	Downregulated	[129]
circ-BPTF	Bromodomain PHD finger transcription factor (BPTF) gene exons 21 to 27	miR-486-5p/CEMIP	2	Chronic obstructive pulmonary disease (COPD)	Human PASMCs	Upregulated	Downregulated	[130]
circ-SWT1 (hsa_circ_0015677)	SWT1 RNA endoribonuclease homolog gene chr1: 185,153,374–185,200,840	miR-192-5p/SOD2	2	H2O2 induced apoptosis in AMI	Human AC16 cardiomyocytes	Downregulated	Upregulated	[131]
circPHKA2 (hsa_circ_0090002)	phosphorylase kinase regulatory subunit alpha 2 (PHKA2) exons 2-29	miR-574-5p/SOD2	2	Acute ischemic stroke (AIS)	Patient blood, immortalized HBMECs	Downregulated	Upregulated	[132]
circRNA_101237	Cyclin-dependent kinase 8 (CDK8) gene exon 10 to exon 12	let-7a-5p/IGF2BP3	2	Anoxia/reoxygenation (A/R) induced cardiomyocyte death	A/R-treated mouse cardiomyocytes	Upregulated	Unaffected by circRNA expression	[133]
circNRG-1	Neuregulin-1 (NRG-1) gene	miR-193b-5p/NRG-1	3	VSMC proliferation in vascular remodeling	Ang-II-treated mouse aortic smooth muscle cells (MASMCs)	Downregulated	Upregulated	[134]

**Table 2:** Consequences of circRNA–protein interactions in cardiovascular diseases.

circRNA-Protein Interaction	circRNA	Interacting Protein	Nature of Interaction	Consequence of Interaction	Cell/Tissue	Physiology/Disease	Refs.
Regulating mRNA stability/expression	Circ-USP9x	ElF4A3	Direct binding in the cytoplasm	Increased stability of GSDMD mRNA, leading to AS-associated pyropstosis	Ox-LDL treated HUVECs	Atherosclerosis	[137]
	CircZNF609	YTHDF3	Direct binding in the cytoplasm	Competitive binding with YAP mRNA to YTHDF3 and modulation of YAP expression to promote heart repair	AC16 human cardiomyocyte	Cardiac repair	[149]
	Autophagy-related circular RNA (ACR mmu_circRNA_006636)	Dnmt3B	Direct binding	Inhibition of DNA methylation of Pink1 promoter, thus blocking the binding of Dnmt3B to suppress cardiac autophagy and I/R injury	Mouse cardiomyocytes	I/R injury of cardiomyocytes in MI	[150]
	CircSMOC1	PTBP1	Direct binding in the nucleus	Competitive inhibition of pyruvate kinase M 1 (PKM1) pre-mRNA to promote the expression of PKM2 and regulate glycolysis	Rat pulmonary artery smooth muscle cells	Pulmonary vascular remodeling and arterial hypertension	[127]
	Circ-TLR4	FUS	Direct binding in the cytoplasm	Promotion of TLR4 mRNA stability in the disease	Human cardiomyocytes	Cardiac hypertrophy	[151]
	CircFndc3b	FUS	Unclear interaction	Interaction with FUS is suggested to regulate FUS mRNA stability and regulate VEGF expression	Rat cardiomyocytes and mouse cardiac endothelial cells	Cardiac repair	[152]
	Circ_SMAD7 (hsa_circ_0000848)	ELAVL1	Unclear interaction	Increased stability of the MI suppressor SMAD7 mRNA, reducing disease effects	HPC-CMs and H9c2 cardiomyocytes	Apoptosis of hypoxia induced cardiomyocytes	[153]
	CircFoxo3	KAT7	Unclear interaction	Suppressing the expression of MI associated factor, HMGB1 by inhibiting KAT7 and reducing the enrichment of H3 lysine acetylation and RNA pol II at the HMGB1 promoter	MI rat model and H9c2 rat cardiomyocytes	MI induced Myocardial injury	[147]
	CircHIPK3	PTEN	Unclear interaction	Decrease in the levels of PTEN mRNA and protein-suppressing cardiomyocyte apoptosis	AC16 human cardiomyocytes	High-glucose-induced cell apoptosis in diabetic cardiomyopathy	[135]
Expression regulation of circRNA by protein	Circ_0029589 (circCHFR)	IFN regulatory factor 1 (IRF1)	m6A modification	Promotion of m6A modifications to the circRNA by IRF1 via m6A methyltransferase METTL3 to downregulate the circRNA in the disease	Human-PBMC-derived macrophages from CAD patients	Acute coronary syndrome and Atherosclerosis	[154]
	Circ_BNIP3 (hsa_circ_0005972)	ElF4A3	Binding to the parent mRNA	ElF4A3 bound to the upstream site of BNIP3 mRNA to induce circBNIP3 expression	Rat H9c2 cells	Hypoxia induced cardiomyocyte injury	[115]
	circSNRK	NOVA alternative splicing regulator 1	Direct binding to flanking introns of pre-circ-SNRK	Inducing circSNRK expression by promoting alternative splicing mediated by the competitive binding of a 55 kDa SNRK peptide	Primary neonatal rat cardiomyocytes and HF rat model	HF and associated hypoxia	[113]
Nuclear translocation or Cytoplasmic sequestration of protein	circHelz	YAP1	Direct binding in the cytoplasm	Promotion of nuclear localization of YAP1 to promote growth and proliferation	Mouse CFs	Cardiac Fibrosis	[143]
	Circ-JA760602	EGR1 and E2F1	Direct binding in the cytoplasm	Inhibited nuclear translocation of EGR1 and E2F1 to suppress transcriptional activation of BCL2	AC16 human cardiomyocytes	Hypoxia induced AMI and associated apoptosis	[138]
	Necroptosis-associated circRNA (CNEACR)	Histone deacetylases 7 (HDAC7)	Direct binding in the cytoplasm	Restriction of the nuclear import of HDAC7 to attenuate FoxA2 transcription and prevent myocardial damage	Mouse cardiomyocytes	Necroptotic death and I/R injury of cardiomyocytes in MI	[155]
	CircEsyt2	PolyC-binding protein 1 (PCBP1)	Direct binding in the cytoplasm	Inhibition of nuclear translocation of PCBP1 to regulate p53 pre-mRNA splicing	Human aortic smooth muscle cells (HASMCs) and mouse VSMCs	Arterial remodeling	[156]
	Circ-Sirt1	Cardiac myosin binding protein-C (c-Myc)	Direct binding in the cytoplasm	Promotion of cytoplasmic sequestration of VSCM-proliferation-associated c-Myc to prevent PDGF-BB-induced binding of c-Myc to the cyclin B1 promoter	Rat VSMCs	Restenosis and neointimal formation after injury	[157]
Protein stability	Ferroptosis-associated circRNA (circFEACR)	NAMPT	Direct binding in the cytoplasm	Increased half-life and stability of NAMPT following cycloheximide treatment without affecting mRNA levels	Mouse cardiomyocytes	Ferroptosis inhibition in myocardial I/R injury	[158]
Protein degradation	CDR1as	MST1	Unclear interaction	Increase in ubiquitination and subsequent degradation of MST1, thus activating Hippo-signaling pathway and promoting apoptosis of cardiomyocytes	Mouse cardiomyocytes	Diabetic cardiomyopathy	[140]
	circNFIX	Y-box binding protein 1 (Ybx1)	Direct binding in the cytoplasm	Promotion of Ybx1 degradation through ubiquitinoylation by competing with E3 ubiquitin ligase Nedd41, influencing the interaction between Ybx1 and Nedd4l	Mouse cardiomyocytes	Cardiac regeneration	[93]
Protein phosphorylation	CircFoxo3	Foxo3	Direct binding	Inhibition of Foxo3 protein phosphorylation at Ser253 via AKT and regulating I/R injury	HL-1 mouse atrial cardiomyocytes	Transplantation induced I/R injury	[139]
Protein complex formation/Protein scaffolds	circYap (hsa_circ_0002320)	Tropomyosin-4 and Gamma-Actin	Direct binding	Enhancing the binding between TPM4 and ACTG to form complexes to inhibit actin polymerization	AC16 human cardiomyocytes and pressure-overload mouse model	Cardiac fibrosis	[141]
	CircHIPK3	HuR and β-TrCP	Direct binding in the cytoplasm	Post-transcriptional regulation and localization of HuR, increasing its association with E3 ubiquitin ligase β-TrCP, promoting its degradation and cardiac senescence	CircHIPK3 KO mouse and mouse cardiomyocytes	Cardiac senescence and aging	[144]
	cZNF292/cZfp292	Syndesmos (SDOS)	Direct binding	Enhancing the interaction between SDC4 and SDOS, thus influencing endothelial cell flow response	Mouse-model-derived aortic endothelium and retinal blood vessel and HUVECs	Endothelial cell morphology	[159]
Mitochondrial recruitment of protein	circSamd4	Vcp protein	Direct binding near the mitochondria	Promotion of mitochondrial localization of Vcp to repress Vdac1 and reactive oxygen species	Mouse fetal and neonatal cardiomyocytes	Cardiac repair and regeneration	[142]
Transcriptional recruitment of protein	circ-RCCD	YY1 transcription factor	Direct binding to YY1 and MyD88 in the cytoplasm	Promoting nuclear localization of YY1 to MyD88 promoter to inhibit its expression and facilitate cell differentiation	Mouse cardiac tissues and cardiomyocyte	Heart development and cardiomyocyte differentiation	[160]
Multifaceted functionality	CircKrt4	Transcriptional activator protein Pur-alpha (Pura)	Direct binding in the nucleus and cytoplasm	Regulation of endothelial-to-mesenchymal transition by modulating the interaction between Pura and N-cadherin	Mouse pulmonary artery endothelial cell (PAEC)	Pulmonary hypertension	[148]
		Glycerol kinase (Glpk)	Direct binding in the nucleus and cytoplasm	Regulating the mitochondrial translocation of Glpk			
		RNA-binding-motif protein 25 (RBM25)	Binding to Krt4 pre-mRNA	Promoting alternative splicing and cyclization of Krt4 gene, thereby inducing circKrt4 expression			

**Table 3:** Functional importance of translated circRNA products.

circRNA	Translated Product	Product Size	Translation Mechanism	Cardiovascular Disease/Physiology	Cell/Tissue/Location	Functional Importance	Refs.
circNlgn	Nlgn173	173aa	Unclear	Cardiac remodeling and cardiac fibrosis	AC16 human cardiomyocytes, Mouse primary cardiomyocytes, CFs and transgenic mouse model	CF growth and cardiomyocyte survival	[164,173]
circ_0036176	Myo9a-208	208aa	IRES	Cardiac remodeling	Human Ac16 cardiomyocytes	Inhibition of CF proliferation through the suppression of cyclin/Rb pathway	[165]
circFNDC3B	circFNDC3B-218aa	218aa	IRES	Unknown	Human CC cell lines	Colon cancer cell proliferation, migration, invasion and epithelial to mesenchymal transition	[152,172]
CircZNF609	ZNF609-250aa	250aa	m6A modification/IRES	Unknown	Mouse and human myoblasts, HeLa, HEK293T	Regulation of myoblast proliferation and ischemic AKI	[179,182,184]
circNPHP4	-	-	Possible IRES	Coronary heart atherosclerotic disease	HCAECs	Unknown	[188]
circ_Lrp6	-	-	ORF detected	Vascular smooth muscle cell activity.	Human VSMCs	Regulation of VSMC activity	[94]
CDR1as	-	-	Suggested	Unknown	Human heart	Unknown	[168]

**Table 4:** CircRNAs as diagnostic biomarkers for cardiovascular disease.

circRNA	Disease/Physiology	Cell/Tissue/Location	Expression	Function	Refs.
circRNA MICRA	LV dysfunction in MI	Human peripheral blood	Low expression	Uncharacterized	[198]
circ 81906-RYR2	AF	Left atrial appendage	Upregulated	Uncharacterized	[199]
hsa_circ_0006314 and hsa_circ_0055387	POAF	Whole blood	Upregulated	Uncharacterized	[200]
circNFAT5	Cardiac arrest	Whole blood	Upregulated	Uncharacterized	[201]
hsa_circ_0000437	Rheumatic valvular heart disease (RVHD)	RVHD plasma samples.	Higher expression	Promotion of cell proliferation and migration, inhibition of apoptosis	[207]
CircSLC8A1	Sudden cardiac death (SCD) caused by acute ischemic heart disease (IHD)	IHD rat and H9c2 cell models	Upregulated	Regulation of I/R injury and cardiomyocyte apoptosis	[109]
circNFIX			Elevated at an early stage of ischemia and later downregulated	Cardiomyocyte apoptosis	
cZNF292	Acute MI	Whole blood, HUVECs, and hESC-CM	Upregulated	Regulation of HUVEC AND hESC-CM apoptosis	[208]
circSMARCA5 (hsa_circ_0001445)	Coronary heart disease (CHD)	Peripheral blood leukocytes	Downregulated	Implicated interaction with several miRNAs	[209]
	Atherosclerosis	Ox-LDL-treated HUVECs	Downregulated	SRSF1/β–catenin pathway influencing cell proliferation	[210]
circCCDC9	Ischemic stroke and I/R injury	Transient middle cerebral artery occlusion (tMCAO) mice model	Decreased	Notch signaling pathway	[211]
circNPHP4	Coronary heart atherosclerotic disease	CAD-related monocytes	Upregulated	Heterogeneous cell adhesion of coronary artery endothelial cells	[188]
circFoxO1	Myopic choroidal vascular dysfunction	RF/6A cells	Upregulated	Inhibition of endothelial effects of angiogenesis	[212]
circFoxo3	Radiation-induced cardiotoxicity and heart damage	AC16 human cardiomyocytes	Upregulated	Protection against cardiotoxicity induced by radiation	[213]
